# Biochemical characterisation of familial hypercholesterolemia: Associations between genetic and lipid profiles

**DOI:** 10.5937/jomb0-62224

**Published:** 2026-01-06

**Authors:** Lukač Sandra Singh, Vladimir Gašić, Jovana Komazec, Ivana Grubiša, Ljiljana Popović, Iva Rasulić, Ana Petakov, Marija Mitrović, Emilija Mihailović, Sonja Pavlović, Katarina Lalić

**Affiliations:** 1 University Clinical Centre of Serbia, Clinic for Endocrinology, Diabetes and Metabolic Disease, Department for Lipid Disorders and Cardiovascular Complications in Diabetes, Belgrade, Serbia; 2 Faculty of Medicine, University of Belgrade; 3 Institute of Molecular Genetics and Genetic Engineering, University of Belgrade; 4 Clinic for Endocrinology, Diabetes and Metabolic Disease, Department for Lipid Disorders and Cardiovascular Complications in Diabetes, University Clinical Centre of Serbia, Belgrade; 5 Emergency Centre, Clinic for the Admission and Management of Emergency Internal Medicine Conditions, Belgrade

**Keywords:** familial hypercholesterolemia, genetics, lipid metabolism, target LDL-C level, familijarna hiperholesterolemija, genetika, metabolizam lipida, ciljni nivo LDL-h

## Abstract

**Background:**

Familial hypercholesterolemia (FH) is characterised by elevated low-density lipoprotein cholesterol (LDL-C) levels and an increased risk of premature cardiovascular disease. The present study aimed to investigate the genetic background, associated biochemical profiles, clinical manifestations, and therapeutic response in patients with clinically suspected FH in Serbia.

**Methods:**

A total of 101 patients with clinically suspected FH were recruited from the Clinic for Endocrinology, Diabetes and Metabolic Diseases in Serbia between 2015 and 2023. Clinical diagnosis was established using the Dutch Lipid Clinic Network (DLCN) criteria. Genetic profiles of all patients were previously determined using next-generation sequencing. Fasting serum lipids, apolipoprotein A-I [ApoA-I], apolipoprotein B [ApoB], and lipoprotein(a) (Lp(a)) were measured enzymatically. Levels of serum lipids were compared between genetically FH-positive (carriers of variants in LDLR, APOB, PCSK9 and LDLRAP1 genes) and FH-negative patients. Therapeutic response was assessed by achieving the LDL-C target level. Statistical analyses were conducted in SPSS (version 30.0).

**Results:**

Genetically confirmed FH patients exhibited significantly higher levels of ApoB (p=0.001) compared with variant-negative individuals, while ApoA-I (p=0.413) and Lp(a) (p=0.421) levels did not differ significantly between groups. Patients with pathogenic FH-associated variants were less likely to reach target LDL-C levels after therapy than those without identified variants.

**Conclusions:**

This study demonstrates biochemical diversity in familial hypercholesterolemia associated with genetic background in the Serbian population. Pathogenic FH mutations were associated with higher ApoB levels, underscoring the importance of combining genetic testing with lipid profiling for precise diagnosis and management.

## Abstract (sr)

**Uvod:**

Familijarna hiperholesterolemija (FH) se karakteriše povišenim nivoima lipoproteina niske gustine (LDL-h) i povećanim rizikom od prevremene kardiovaskularne bolesti. Cilj ove studije bio je da ispita genetsku osnovu, povezane biohemijske profile, kliničke manifestacije i terapijski odgovor kod pacijenata sa kliničkom sumnjom na FH u Srbiji.

**Metode:**

Obuhvaćen je 101 pacijent sa kliničkom sumnjom na FH, koji su lečeni u periodu od 2015. do 2023. godine na Klinici za endokrinologiju, dijabetes i bolesti metabolizma u Srbiji. Klinička dijagnoza je postavljena korišćenjem kriterijuma Holandske mreže lipidnih klinika (DLCN). Genetski profili svih pacijenata su određeni primenom metode sekvenciranja nove generacije. Serumski lipidni parametri naste, apolipoprotein A-I [ApoA-I], apolipoprotein B [ApoB] i lipoprotein(a) (Lp(a)) određivani su enzimskim metodama. Nivoi serumskih lipida poređeni su između genetski FH-pozitivnih pacijenata (nosioca varijanti u genima LDLR, APOB, PCSK9 i LDLRAP1) i FH-negativnih pacijenata. Terapijski odgovor je procenjivan na osnovu postizanja ciljnog nivoa LDL-h. Statističke analize su sprovedene u SPSS-u (verzija 30.0).

**Rezultati:**

Genetski potvrđeni FH pacijenti imali su značajno više nivoe ApoB (p = 0.001) u poređenju sa pacijentima bez identifikovanih varijanti, dok se nivoi ApoA-I (p = 0.413) i Lp(a) (p = 0.421) nisu značajno razlikovali između grupa. Pacijenti sa patogenim FH-povezanim varijantama ređe su dostizali ciljne LDL-h vrednosti nakon terapije u poređenju sa onima bez identifikovanih varijanti.

**Zaključak:**

Ova studija pokazuje biohemijsku raznolikost porodične hiperholesterolemije povezanu sa genetskim karakteristikama u populaciji Srbije. Patogene FH mutacije bile su povezane sa višim nivoima ApoB, što naglašava značaj kombinovanja genetskog testiranja i lipidnog profilisanja za preciznu dijagnozu i adekvatno lečenje.

## Introduction

Familial hypercholesterolemia (FH) is one of the most common monogenic disorders of lipid metabolism, characterised by markedly elevated plasma low-density lipoprotein cholesterol (LDL-C) levels and a high risk of premature atherosclerotic cardiovascular disease (ASCVD) [1] [2]. The estimated prevalence of heterozygous FH is approximately 1 in 250 individuals worldwide, while the homozygous form occurs in about 1 in 300,000 to 1,000,000 [3]. Despite this relatively high frequency, FH remains underdiagnosed and undertreated in most populations, leading to substantial preventable cardiovascular morbidity and mortality [4]. The disorder is primarily caused by pathogenic variants in genes regulating low-density lipoprotein (LDL) metabolism, most commonly in the *LDLR* (low-density lipoprotein receptor), *APOB* (apolipoprotein B), and *PCSK9* (proprotein convertase subtilisin/kexin type 9) genes [5] [6] [7]. Less frequently, mutations in *LDLRAP1* (LDLR adaptor protein 1) lead to an autosomal recessive form of FH [8]. Defects in *LDLR* reduce clearance of circulating LDL particles, *APOB* variants impair receptor binding, and *PCSK9* gain-of-function pathogenic variants increase receptor degradation - all resulting in elevated plasma low-density lipoprotein cholesterol (LDL-C) level [9] [10]. The biochemical hallmark of FH is thus persistent, lifelong hypercholesterolemia, often accompanied by elevated apolipoprotein B (apoB) and lipoprotein(a) (Lp(a)). Triglyceride levels vary according to genotype and metabolic background [11] [12]. The genetic and phenotypic heterogeneity explain the broad clinical spectrum among FH patients, from asymptomatic hyperlipidemia to premature ASCVD, even within the same family or same variant type [13]. Diagnosis of FH is based on fasting serum lipid parameters, clinical (physical) findings and genetic testing. Previous studies have demonstrated that carriers of *LDLR* mutations tend to exhibit higher LDL-C levels and earlier onset of ASCVD compared to those with *APOB* or *PCSK9* variants [14]. However, other contributing factors, such as polygenic background, dietary habits, hormonal status, and variants in other lipid-modifying genes, can modulate lipid profiles and disease severity [15] [16]. Understanding these genotype-phenotype relationships is crucial for accurate diagnosis, risk stratification, and selection of appropriate therapy [17]. The broader implementation of genetic testing, such as NGS (next-generation sequencing), now allows comprehensive identification of FH-associated variants and facilitates correlation of genetic findings with biochemical and clinical parameters [18].

Evaluating lipid and apolipoprotein patterns alongside genetic results provides deeper insight into the molecular mechanisms underlying dyslipidemia and facilitates precision medicine approaches for the management of FH [4]. The present study aims to investigate the relationship between genetic variants and several biochemical lipid parameters in patients with clinically diagnosed familial hypercholesterolemia. By analysing lipid and apolipoprotein profiles across different genotype groups, the study seeks to elucidate genotype-phenotype correlations and highlights the biochemical consequences of specific molecular defects associated with FH.

## Materials and methods

### Patient cohort

This study included patients with clinical suspicion of familial hypercholesterolemia (FH) who were diagnosed according to the criteria of the Dutch Lipid Clinic Network (DLCN). The cohort comprised 101 patients (94 unrelated individuals and 7 affected relatives) evaluated between 2015 and 2023 at the Unit for Lipid Disorders, Clinic for Endocrinology, Diabetes and Metabolic Diseases in Serbia. General practitioners or cardiologists referred participants for specialised assessment. Demographic, clinical, biochemical and genetic data obtained in a previous study were further analysed to explore additional lipid-related parameters in relation to genetic status [19]. Inclusion criteria required elevated LDL-C levels accompanied by a personal or family history of premature cardiovascular disease and/or phenotypic signs (tendon xanthomas, xanthelasmas, or corneal arcus). Individuals with secondary hyperlipidemia and those using lipid-altering drugs were excluded. Genetic screening for pathogenic variants in FH-associated genes had been completed previously [19].

The study protocol was approved by the Research Ethics Committee of the Institute of Molecular Genetics and Genetic Engineering, University of Belgrade (approval number O-EO-059/2024). Written informed consent was obtained from all participants before enrollment.

### Laboratory analyses

Fasting serum lipid parameters, including apo- lipoprotein A-I (ApoA-I) and apolipoprotein B (ApoB), were quantified enzymatically using a commercially available kit (Boehringer Mannheim GmbH Diagnostica, Germany). Low-density lipoprotein cholesterol (LDL-C) concentrations were calculated using the Friedewald formula. Lipoprotein(a) (Lp(a)) concentrations were determined by an immunoturbidimetric assay using an automated analyser (Cobas 8000, Roche Diagnostics, Mannheim, Germany). The assay was calibrated according to the manufacturer's protocol, and results were expressed in mg/dL [20].

### Diagnostic criteria for familial hypercholesterolemia

The diagnosis of FH was established according to the Dutch Lipid Clinic Network (DLCN) scoring system, which integrates lipid concentrations, clinical manifestations, family history, LDL-C concentration, and physical examination findings into a composite score. Based on the total DLCN score, patients were categorised as having a definite FH diagnosis (score >8), probable FH (score 6-8), possible FH (score 3-5), or unlikely FH. All diagnostic categories were included in the analysis [21].

### Definition of cardiovascular events and LDL-C targets

Achievement of target LDL-cholesterol levels was evaluated according to current European Society of Cardiology (ESC) and European Atherosclerosis Society (EAS) guidelines for the management of dyslipidemias [22]. The LDL-C target was set at <3.0 mmol/L for patients without a history of cardiovascular (CV) events and at <1.8 mmol/L for those with documented CV disease. Based on these thresholds, a binary variable (LDL target) was created to indicate whether each patient achieved the LDL target following lipid-lowering therapy (1 = target achieved; 0 = target not achieved). The presence of CV events, including myocardial infarction, angina pectoris, coronary revascularisation, or stroke, was determined from medical history and clinical documentation.

### Statistical analysis

All statistical analyses were conducted using IBM SPSS Statistics for Windows, Version 30.0 (IBM Corp., Armonk, NY, USA). Data distribution was evaluated with the Shapiro-Wilk test, which demonstrated non-normality for all continuous variables (p<0.05). Therefore, non-parametric tests were applied in all analyses. Continuous variables were expressed as median and interquartile range (IQR), and categorical variables were presented as absolute frequencies and percentages. Comparison between two independent groups was performed using the Mann-Whitney U test, and differences in categorical variables were assessed using the Chi-square test ( [). All tests were two-tailed, and p-values <0.05 were considered statistically significant. Data visualisation and descriptive statistics were generated using the same software.

## Results

### Study population

The study included a total of 101 patients clinically suspected of familial hypercholesterolemia (FH). The mean age of participants was 55.1±15.8 years. The mean total cholesterol (TC) concentration (7.83±2.84 mmol/L), the mean low-density lipoprotein cholesterol (LDL-C) level (5.41 ±2.22 mmol/L), the mean high-density lipoprotein cholesterol (h DL-C) (1.32±0.34 mmol/L), and the mean triglyceride (TG) level (1.89±1.11 mmol/L) were previously determined [19]. Among participants with available apolipoprotein data, the mean apolipoprotein A-I (ApoA-I) concentration was 1.60±0.37 g/L (n = 88), and the mean apolipoprotein B (ApoB) concentration was 1.44±0.55 g/L (n=90). The mean lipoprotein(a) (Lp(a)) concentration was 0.31 ±0.37 mg/dL (n = 98) (Table 1). Physical manifestations of FH, including tendon xanthomas, xanthelasmas, and corneal arcus, were observed in 11 patients (10.9%).

**Table 1:** Descriptive statistics of lipid and apolipoprotein parameters in the study population. Note: Values for TC, LDL-C, HDL-C and TG were previously reported [19]. N - number of patients. Data are presented as mean±SD.

Parameter	N	Minimum	Maximum	Mean±SD	Units
Total cholesterol (TC) [19]	101	2.35	19.00	7.83±2.84	mmol/L
LDL-C [19]	101	1.32	12.00	5.41±2.22	mmol/L
HDL-C [19]	101	0.52	2.68	1.32±0.34	mmol/L
Triglycerides (TG) [19]	101	0.41	8.26	1.89±1.11	mmol/L
Lipoprotein(a) [Lp(a)]	98	0.024	1.560	0.31±0.37	g/L
Apolipoprotein A-I (ApoA-I)	88	0.83	3.48	1.60±0.37	g/L
Apolipoprotein B (ApoB)	90	0.33	2.70	1.44±0.55	g/L

### Lipid and apolipoprotein parameters according to genetic status in FH patients

**Figure 1:**
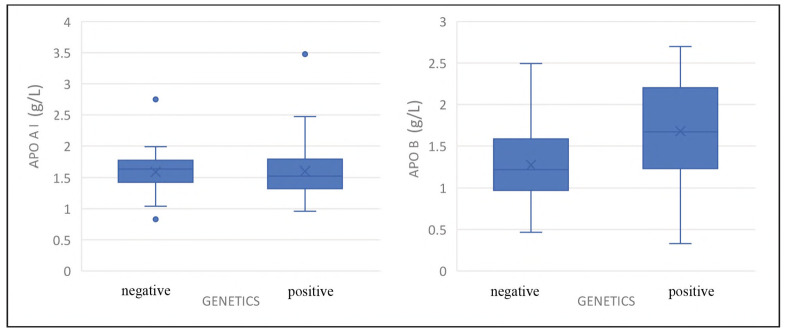
Distribution of apolipoprotein A-I (ApoA-I) and apolipoprotein B (ApoB) concentrations in patients with (positive) and without (negative) genetically confirmed familial hypercholesterolemia (FH). Data are presented as boxplots showing median, interquartile range, and outliers.

All 101 patients underwent genetic analysis for variants in the four key genes implicated in familial hypercholesterolemia: *LDLR, APOB, PCSK9*, and *LDLRAP1*. Previously, we identified pathogenic variants in 44 out of 101 patients (43.6%), with the majority located in the LDLR gene (93.2% of all genetically positive patients) [19]. To explore the impact of genetic background on lipid metabolism, ApoA-I, ApoB, and Lp(a) were compared between FH-positive and FH-negative patients with genetic confirmation. No significant difference in ApoA-I levels was observed between the two groups (p = 0.413) (Figure 1). In contrast, apolipoprotein B (ApoB) levels were significantly higher among mutation carriers compared with non-carriers (p=0.001) (Figure 1). Additionally, difference in Lp(a) levels between the two groups was not detected (p=0.421).

### LDL-C target values

Analysis of the therapeutic response showed that patients with genetically confirmed familial hypercholesterolemia (FH) achieved a greater percentage reduction in LDL cholesterol than those without identified genetic variants (p=0.017). However, the absolute post-treatment LDL-C concentrations remained higher in genetically positive patients (p=0.043), consistent with the expected more severe phenotype. A statistically significant association was observed between genetic status and achieving LDL-C target values (p=0.004). Patients with pathogenic FH-associated variants were less likely to reach target LDL-C levels after therapy than those without identified genetic variants (Table 2).

**Table 2:** Association between genetic status and achievement of LDL-C treatment targets. Note: Target LDL-C was defined as <3.0 mmol/L for patients without prior CV events and <1.8 mmol/L for those with documented CV disease. N - number of patients.

Genetic status	N	Target<br>achieved<br>N (%)	Target not<br>achieved<br>N (%)	Total<br>N (%)
Pathogenic genetic variants present	36	8 (22.2%)	28 (77.8%)	36 (100%)
No identified genetic variants	65	31 (47.7%)	34 (52.3%)	65 (100%)
** Total **	** 101 **	** 39 (38.6%) **	** 62 (61.4%) **	** 101 (100%) **

## Discussion

This study provides biochemical characterisation and the relationship between genetic variants and several lipid parameters in patients with clinically suspected familial hypercholesterolemia (FH) from a Serbian cohort. Additionally, clinical manifestations and therapeutic response were analysed in the same context. Using next-generation sequencing (NGS), pathogenic or likely pathogenic variants in the *LDLR, APOB*, and *PCSK9* genes were identified in 43.6% of patients [19]. These findings are consistent with mutation detection rates reported in other European populations, which typically range between 30% and 60%, depending on the inclusion criteria and sequencing methodology used [23] [24] [25]. The overall mutation detection rate of 43.6% supports the clinical utility of genetic testing in patients meeting DLCN diagnostic criteria. Notably, as previously observed, a substantial proportion of genetically positive cases were found among patients classified as »possible« or even »unlikely« FH by the DLCN score, indicating that reliance on clinical scoring systems alone may underestimate the true prevalence of molecularly confirmed FH [19]. This emphasises the value of incorporating molecular diagnostics into standard clinical workflows to improve accuracy and cascade. In patients with genetically confirmed familial hypercholesterolemia (FH), lipid profiles showed pronounced elevations in LDL-C and total cholesterol, consistent with the well-established biochemical pathophysiology of the disorder [19]. As expected, genetically confirmed FH patients exhibited significantly higher LDL-C and total cholesterol levels than those without identified genetic variants. These findings reflect impaired clearance of low-density lipoprotein particles due to defective receptor-mediated uptake, a hallmark of carriers of LDLR and APOB variants. Moreover, gain-of-function variants in *PCSK9* can exacerbate this phenotype by promoting LDL receptor degradation, resulting in further increases in plasma LDL-C [1] [5] [9] [10]. In keeping with these observations, our genetically FH-positive cohort not only showed significantly higher LDL-C and total cholesterol levels than non-variant carriers (p<0.001), but also displayed a persistent post-therapy LDL elevation, suggesting an entrenched genetic »set point« in lipoprotein metabolism. Recent studies reinforce the notion that variant class matters: carriers of null LDLR alleles generally exhibit markedly higher LDL-C and greater atherosclerotic risk than those with less damaging (receptor-defective) alleles, and the gradient of LDL-C elevation correlates with residual receptor function. In a comprehensive meta-analysis, patients with null variants had more severe phenotypes and higher LDL-C levels than those with receptor-defective variants or non-LDLR variant carriers [26] [27] [28]. Moreover, genotype-specific LDLR analyses using variant-adjusted percentile frameworks have recently been proposed better to stratify cardiovascular risk among FH patients [29]. Even with modern lipid-lowering therapy, many patients with genetically confirmed FH fail to reach LDL-C targets, a finding consistent across real-world cohorts. For example, in clinical practice settings, PCSK9 inhibitors among FH patients have yielded LDL-C reductions between 40-60%, yet many still do not achieve guideline-specified goals [30] [31] [32] [33]. These residual elevations mirror the more severe post-therapy LDL-C levels we observed in genetic variant carriers and highlight the need for tailored intensified regimens, especially in high-risk FH genotypes that may require combination approaches or novel therapies. Although HDL-C concentrations did not differ between variant carriers and non-carriers in our cohort [19], this is consistent with the FH literature, which shows that HDL-C is often preserved in heterozygous FH and is not a defining biochemical feature of the disorder. Several FH series and reviews report no consistent HDL-C decrement compared with non-FH populations, despite marked LDL-C elevation [34]. In this cohort, triglyceride (TG) concentrations were modest but significantly higher in patients with genetically confirmed familial hypercholesterolemia (FH) than in those without identified variants. Although hypertriglyceridemia is not a defining feature of FH, recent data suggest that secondary genetic and metabolic modifiers can contribute to TG elevation even in monogenic FH [35] [36]. Polygenic TG burden, insulin resistance, adiposity, and hepatic overproduction of VLDL may coexist with *LDLR, APOB*, or *PCSK9* gene variants, producing a more atherogenic »mixed« lipid phenotype [36]. Mechanistically, elevated TG reflects delayed clearance of triglyceride-rich lipoproteins (TRLs) and remnants, influenced by regulators such as apoC-III and ANGPTL3, which inhibit lipoprotein lipase and remnant catabolism [37] [38]. Moreover, ANGPTL3 inhibition (e.g., evinacumab) effectively reduces TG and LDL-C in FH and severe dyslipidemia, reinforcing the therapeutic relevance of this pathway [39] [40] [41]. In this study, apolipoprotein A-I (ApoA-I) concentrations did not differ significantly between patients with and without genetically confirmed FH, indicating that HDL metabolism remains largely unaffected by classical FH genetic variants. This finding is consistent with previous reports showing that *LDLR, APOB*, and *PCSK9* variants predominantly alter low-density lipoprotein metabolism, while HDL-related pathways are preserved [10] [13]. Minor variability in ApoA-I levels among FH cohorts has been attributed more to metabolic status and lifestyle factors than to underlying genotype [10] [42]. Conversely, ApoB levels were significantly higher in variant carriers, reflecting the central role of ApoB-containing lipoproteins in the FH phenotype. ApoB represents the structural protein of LDL, VLDL, and Lp(a), and its concentration directly reflects the number of circulating atherogenic particles rather than cholesterol mass alone [43] [44]. Elevated ApoB is therefore a robust biochemical hallmark of FH, frequently showing stronger associations with ASCVD risk than LDL-C levels, particularly in patients with discordant LDL-C and ApoB values [45] [46]. Current European and international lipid guidelines increasingly emphasise ApoB as an adjunct or alternative risk marker for monitoring treatment response in FH [47]. In contrast, Lp(a) concentrations did not differ significantly between FH patients with and without a genetic risk factor. This observation aligns with contemporary data suggesting that Lp(a) levels are determined by independent genetic variation at the *LPA* locus, rather than by mutations in *LDLR, APOB*, or *PCSK9* [48]. Elevated Lp(a) frequently coexists with FH, leading to a »double genetic hit« that further increases cardiovascular risk [49] [12]. Although the two traits are genetically distinct, their combined presence identifies patients at particularly high risk of premature ASCVD, warranting intensified lipid-lowering and emerging Lp(a)-targeted therapies (e.g., antisense oligonucleotides and siRNA-based approaches) [12] [50]. Overall, these findings reinforce the complementary roles of ApoB and Lp(a) as key atherogenic markers in FH, while highlighting that ApoA-I remains largely unaffected by classical FH genetic variants. Comprehensive lipoprotein profiling, including ApoB and Lp(a) measurement, thus improves phenotypic characterisation and enhances cardiovascular risk stratification in genetically defined FH.

Patients in our cohort with genetically confirmed FH demonstrated a greater proportional reduction in LDL-cholesterol following therapy compared with those without identified pathogenic variants. Despite this relative improvement, absolute post-treatment LDL-C levels remained higher in genetically confirmed patients, reflecting the more severe lipid phenotype typically associated with FH. These observations suggest that such patients, particularly those carrying pathogenic variants in LDLR, APOB, or PCSK9, may require more intensive or combination lipid-lowering regimens to achieve optimal LDL-C control. These findings are consistent with previous studies showing that, despite intensive lipid-lowering therapy, many FH patients, particularly those with receptor-negative variants, fail to achieve recommended LDL-C targets (<1.8 mmol/L for secondary prevention or <1.4 mmol/L in very-high-risk individuals) [22] [17] [51]. Contemporary European and international guidelines emphasise that most genetically confirmed FH patients require combination therapy, typically high-intensity statins plus ezetimibe and often PCSK9 inhibitors, to approach target levels [2] [52]. Even with such a regimen, real-world studies reveal that a substantial proportion remains above therapeutic goals [53]. The magnitude of LDL-C reduction is influenced by both the functional class of the LDLR variant (defective versus null,) and adherence to therapy, while newer LDLR-independent agents such as evinacumab (ANGPTL3 inhibition) offer additional benefit in severe phenotypes, including homozygous FH [40]. Collectively, these data underscore the genetic determinants of treatment response in FH and the ongoing need for intensified, mechanism-targeted therapies.

The present study reinforces the strong association between the presence of FH-causing genetic variants and the severity of hypercholesterolemia before therapy. Patients with genetically confirmed FH are at markedly higher cardiovascular risk, often requiring earlier and more aggressive lipid-lowering interventions. The identification of specific variants, such as *LDLR* c.858C>A (p.Ser286Arg), which in this cohort was associated with relatively lower LDL-C levels [19], may provide insight into variant-specific functional effects and potential differences in response to therapy. These genotype-phenotype correlations have significant clinical implications. Genetic testing not only confirms diagnosis, but also guides cascade screening in families, enabling early detection and treatment. Moreover, genetic information supports a precision medicine approach, as patients with *LDLR*-negative variants often respond differently to statins and may benefit from PCSK9 inhibitors or combination therapy [2] [4] [17] [52]. The results of this study are in line with several European FH registries, including those from the Netherlands, Spain, and the UK, which have reported similar mutation spectra and lipid profiles among affected individuals [54] [55] [56]. However, some regional differences exist, likely reflecting population-specific founder mutations and genetic heterogeneity. The inclusion of a large proportion of patients with »possible« FH by clinical criteria but positive genetic results highlight the limitations of the DLCN score, particularly in regions where phenotypic expression may vary due to environmental or metabolic modifiers.

## Conclusion

In conclusion, this study demonstrates that patients with genetically confirmed familial hypercholesterolemia present with a more severe biochemical phenotype, characterised by markedly elevated lipid concentrations before therapy. The observed association between pathogenic variants and lipid abnormalities highlights the clinical importance of incorporating genetic testing into the diagnostic and therapeutic framework for FH. By integrating molecular, clinical, and biochemical data, a more precise understanding of disease mechanisms can be achieved, enabling personalised risk assessment, optimised treatment strategies, and effective cascade screening within affected families.

## Dodatak

### Acknowledgements

This study was supported by a project of the Ministry of Science, Technological Development and Innovation, the Republic of Serbia (EN 451-03-136/2025-03/200042), and unrestricted support by Novartis (IIT-87939941).

### Conflict of interest statement

All the authors declare that they have no conflict of interest in this work.

### List of abbreviations

FH, familial hypercholesterolemia;<br>LDL-C, low-density lipoprotein cholesterol;<br>HDL-C, high-density lipoprotein cholesterol;<br>TC, total cholesterol;<br>TG, triglycerides;<br>ApoA-I, apolipoprotein A-I;<br>ApoB, apolipoprotein B;<br>Lp(a), lipoprotein(a);<br>DLCN, Dutch Lipid Clinic Network
